# Association between birthweight and later body mass index: an individual-based pooled analysis of 27 twin cohorts participating in the CODATwins project

**DOI:** 10.1093/ije/dyx031

**Published:** 2017-03-19

**Authors:** Aline Jelenkovic, Yoshie Yokoyama, Reijo Sund, Kirsi H Pietiläinen, Yoon-Mi Hur, Gonneke Willemsen, Meike Bartels, Toos CEM van Beijsterveldt, Syuichi Ooki, Kimberly J Saudino, Maria A Stazi, Corrado Fagnani, Cristina D’Ippolito, Tracy L Nelson, Keith E Whitfield, Ariel Knafo-Noam, David Mankuta, Lior Abramson, Kauko Heikkilä, Tessa L Cutler, John L Hopper, Jane Wardle, Clare H Llewellyn, Abigail Fisher, Robin P Corley, Brooke M Huibregtse, Catherine A Derom, Robert F Vlietinck, Ruth JF Loos, Morten Bjerregaard-Andersen, Henning Beck-Nielsen, Morten Sodemann, Adam D Tarnoki, David L Tarnoki, S Alexandra Burt, Kelly L Klump, Juan R Ordoñana, Juan F Sánchez-Romera, Lucia Colodro-Conde, Lise Dubois, Michel Boivin, Mara Brendgen, Ginette Dionne, Frank Vitaro, Jennifer R Harris, Ingunn Brandt, Thomas Sevenius Nilsen, Jeffrey M Craig, Richard Saffery, Finn Rasmussen, Per Tynelius, Gombojav Bayasgalan, Danshiitsoodol Narandalai, Claire MA Haworth, Robert Plomin, Fuling Ji, Feng Ning, Zengchang Pang, Esther Rebato, Robert F Krueger, Matt McGue, Shandell Pahlen, Dorret I Boomsma, Thorkild IA Sørensen, Jaakko Kaprio, Karri Silventoinen

**Affiliations:** 1 Department of Social Research, University of Helsinki, Helsinki, Finland; 2 Department of Genetics, Physical Anthropology and Animal Physiology, University of the Basque Country UPV/EHU, Leioa, Spain; 3 Department of Public Health Nursing, Osaka City University, Osaka, Japan; 4 Obesity Research Unit, Research Programs Unit, University of Helsinki, Helsinki, Finland and Abdominal Center, Endocrinology, Helsinki University Central Hospital, Helsinki, Finland; 5 Department of Education, Mokpo National University, Jeonnam, South Korea; 6 Department of Biological Psychology, VU University Amsterdam, Amsterdam, The Netherlands; 7 Department of Health Science, Ishikawa Prefectural Nursing University, Kahoku, Ishikawa, Japan; 8 Boston University, Department of Psychological and Brain Sciencies, Boston, MA, USA; 9 Istituto Superiore di Sanità—National Center for Epidemiology, Surveillance and Health Promotion, Rome, Italy; 10 Department of Health and Exercise Sciencies and Colorado School of Public Health, Colorado State University, USA; 11 Psychology and Neuroscience, Duke University, Durham, NC, USA; 12 The Hebrew University of Jerusalem, Jerusalem, Israel; 13 Hadassah Hospital Obstetrics and Gynecology Department, Hebrew University Medical School, Jerusalem, Israel; 14 Department of Public Health, University of Helsinki, Helsinki, Finland; 15 The Australian Twin Registry, Centre for Epidemiology and Biostatistics, The University of Melbourne, Melbourne, Victoria, Australia; 16 Department of Epidemiology, School of Public Health, Seoul National University, Seoul, Korea; 17 Health Behaviour Research Centre, Department of Epidemiology and Public Health, Institute of Epidemiology and Health Care, University College London, London, UK; 18 Institute for Behavioral Genetics, University of Colorado, Boulder, Colorado, USA; 19 Centre of Human Genetics, University Hospitals Leuven, Leuven, Belgium; 20 Department of Obstetrics and Gynaecology, Ghent University Hospitals, Ghent, Belgium; 21 The Charles Bronfman Institute for Personalized Medicine, The Mindich Child Health and Development Institute, Icahn School of Medicine at Mount Sinai, New York, NY, USA; 22 Bandim Health Project, INDEPTH Network, Bissau, Guinea-Bissau; 23 Research Center for Vitamins and Vaccines, Statens Serum Institute, Copenhagen, Denmark; 24 Department of Endocrinology, Odense University Hospital, Odense, Denmark; 25 Department of Infectious Diseases, Odense University Hospital, Odense, Denmark; 26 Department of Radiology and Oncotherapy, Semmelweis University, Budapest, Hungary; 27 Hungarian Twin Registry, Budapest, Hungary; 28 Michigan State University, East Lansing, Michigan, USA; 29 Department of Human Anatomy and Psychobiology, University of Murcia, Murcia, Spain; 30 IMIB-Arrixaca, Murcia, Spain; 31 Department of Developmental and Educational Psychology, University of Murcia, Murcia, Spain; 32 QIMR Berghofer Medical Research Institute, Brisbane, Australia; 33 School of Epidemiology, Public Health and Preventive Medicine, University of Ottawa, Ottawa, Ontario, Canada; 34 School of Psycholoy, Laval University, Quebec, Canada; 35 Institute of Genetic, Neurobiological, and Social Foundations of Child Development, Tomsk State University, Russian Federation; 36 Departement of Psychology, University of Quebec at Montreal, Montreal, Quebec, Canada; 37 School of Psychoeducation, University of Montreal, Montreal, Quebec, Canada; 38 Norwegian Institute of Public Health, Oslo, Norway; 39 Murdoch Childrens Research Institute, Royal Children’s Hospital, Parkville, Victoria, Australia; 40 Department of Paediatrics, University of Melbourne, Parkville, Victoria, Australia; 41 Department of Public Health Sciences, Karolinska Institutet, Stockholm, Sweden; 42 Healthy Twin Association of Mongolia, Ulaanbaatar, Mongolia; 43 Graduate School of Biomedical and Health Sciences, Hiroshima University, Hiroshima, Japan; 44 MRC Integrative Epidemiology Unit, University of Bristol, Bristol, UK; 45 King’s College London, MRC Social, Genetic & Developmental Psychiatry Centre, Institute of Psychiatry, Psychology & Neuroscience, London, UK; 46 Department of Noncommunicable Diseases Prevention, Qingdao Centers for Disease Control and Prevention, Qingdao, China; 47 Department of Psychology, University of Minnesota, Minneapolis, MN, USA; 48 Novo Nordisk Foundation Centre for Basic Metabolic Research (Section on Metabolic Genetics), and Department of Public Health, Faculty of Health and Medical Sciences, University of Copenhagen, Copenhagen, Denmark; 49 Department of Clinical Epidemiology (formely Institute of Preventive Medicine), Bispebjerg and Frederiksberg Hospitals, Copenhagen, The Capital Region, Denmark; 50 National Institute for Health and Welfare, Helsinki, Finland; 51 Institute for Molecular Medicine FIMM, Helsinki, Finland; 52 Osaka University Graduate School of Medicine, Osaka University, Osaka, Japan

**Keywords:** birthweight, body mass index, twins

## Abstract

**Background:**

There is evidence that birthweight is positively associated with body mass index (BMI) in later life, but it remains unclear whether this is explained by genetic factors or the intrauterine environment. We analysed the association between birthweight and BMI from infancy to adulthood within twin pairs, which provides insights into the role of genetic and environmental individual-specific factors.

**Methods:**

This study is based on the data from 27 twin cohorts in 17 countries. The pooled data included 78 642 twin individuals (20 635 monozygotic and 18 686 same-sex dizygotic twin pairs) with information on birthweight and a total of 214 930 BMI measurements at ages ranging from 1 to 49 years. The association between birthweight and BMI was analysed at both the individual and within-pair levels using linear regression analyses.

**Results:**

At the individual level, a 1-kg increase in birthweight was linearly associated with up to 0.9 kg/m^2^ higher BMI (*P* < 0.001). Within twin pairs, regression coefficients were generally greater (up to 1.2 kg/m^2^ per kg birthweight, *P* < 0.001) than those from the individual-level analyses. Intra-pair associations between birthweight and later BMI were similar in both zygosity groups and sexes and were lower in adulthood.

**Conclusions:**

These findings indicate that environmental factors unique to each individual have an important role in the positive association between birthweight and later BMI, at least until young adulthood.

**Key Messages**

- Birthweight is positively and linearly associated with later body mass index (BMI).
- The association between birthweight and BMI from infancy onwards is similar in males and females, and is lower in adulthood.
- Environmental factors unique to each individual have an important role in the positive association between birthweight and later BMI.

## Introduction

The increasing prevalence of overweight and obesity over the last decades has grown into a global epidemic that currently affects a large part of the world’s population.^1^ The interest in the role of gestational factors behind adult health outcomes^2^ has resulted in a number of epidemiological studies analysing the association between birthweight and later body mass index (BMI). Several very large and well-conducted studies have shown a positive association of birthweight with BMI and overweight/obesity in children, adolescents and adults,^3–9^ but J- or U-shaped associations have also been reported.^10^^,^^11^ The mechanisms underlying this association are, however, still poorly understood. It has been suggested that the fetal period may be critical for the development of obesity,^10^^,^^12^ but it is unclear how far the associations between birthweight and subsequent BMI reflect early developmental factors in the intrauterine environment or whether they are explained by common genetic factors affecting body size from fetal life until adulthood.

Twins create a natural experiment and offer an opportunity to shed light on the mechanisms underlying the association between birth and later BMI.^13^^,^^14^ Twins come from the same family, share the same maternal environment, have the same gestational age and, in the case of monozygotic (MZ) twins, are genetically identical. However, each fetus has its own fetoplacental environmental conditions, such as supply of nutrients and oxygen, which may differ substantially from that of its co-twin.^15^ The association between the intra-pair differences in birthweight and later BMI cannot be explained by shared family factors, such as maternal nutrition, parental education or socio-economic status. Further, differences within MZ pairs cannot be explained by preconceptional parental influences or genetic factors. The comparison of intra-pair associations in MZ and dizygotic (DZ) twins is thus a strong design to explore within family effects. A stronger association in DZ than in MZ twins is taken as evidence that the relationship between birthweight and later BMI is explained by genetic factors. Differences in birthweight and later BMI within MZ pairs can only be influenced by environmental factors that are unique to individuals (i.e. the intrauterine environment), whereas differences within DZ pairs can also be influenced by genetic factors.^13^^,^^14^

A few twin studies have performed pair-wise analyses between birthweight and BMI in late adolescence and adulthood, but the results have been somewhat conflicting. Intra-pair differences in birthweight were not related with intra-pair differences in BMI in adults from the USA (Minnesota) and the UK.^16^^,^^17^ In young adult Belgian MZ twins, only when the birthweight difference between the twins exceeded 15%, the heavier twin at birth showed a trend towards a higher BMI.^18^^,^^19^ A positive association was observed in Swedish young adult MZ males^20^ and in Finnish MZ and DZ twins of both sexes (aged 16–18.5 years).^21^ This suggests that intrauterine environment may play a role in later BMI, but this is far from settled. Moreover, it is not known whether the effects vary in their importance by age, particularly in childhood. To address these questions, we analysed the association between birthweight and later BMI from infancy to adulthood in MZ and DZ twins of both sexes in multinational twin data from 27 cohorts in 17 countries.

## Material and methods

### Sample

This study is based on the data from the COllaborative project of Development of Anthropometrical measures in Twins (CODATwins), which was intended to pool data from all twin projects in the world having information on height and weight.^22^ Information on birthweight was available in 27 cohorts; birth length and gestational age were available in 14 and 17 of these cohorts, respectively. The participating twin cohorts are identified in Table 1 (footnote) and were previously described in detail.^22^

**Table 1:** Descriptive statistics of birthweight and BMI by zygosity, age and sex

	Males						Females					
	
	MZ			DZ			MZ			DZ		
	*N*	Mean	SD	*N*	Mean	SD	*N*	Mean	SD	*N*	Mean	SD
Birthweight (kg)	19 864	2.52	0.55	19 208	2.60	0.57	21 406	2.41	0.52	18 164	2.50	0.54
**BMI (kg/m^2^)**												
Age 1	5572	17.15	1.41	5070	17.11	1.35	5966	16.78	1.41	4692	16.71	1.34
Age 2	4448	16.54	1.39	4212	16.53	1.43	4540	16.09	1.37	3666	16.15	1.36
Age 3	5490	15.94	1.37	5298	15.96	1.50	6176	15.61	1.43	4968	15.68	1.54
Age 4	3042	15.85	1.75	2950	15.93	1.86	3152	15.65	1.95	2750	15.69	1.87
Age 5	2488	15.25	1.52	2342	15.29	1.61	2678	15.06	1.60	2078	15.18	1.72
Age 6	1058	15.43	1.73	660	15.47	1.89	922	15.18	1.68	530	15.32	2.22
Age 7	4536	15.34	1.68	3954	15.43	1.89	5018	15.36	1.90	3826	15.46	2.01
Age 8	2066	15.57	1.64	1494	15.72	2.01	2078	15.55	1.90	1264	15.79	2.09
Age 9	1982	16.24	2.07	1466	16.52	2.48	2008	16.24	2.33	1290	16.50	2.66
Age 10	3776	16.56	2.21	3184	16.59	2.32	4074	16.59	2.40	2892	16.79	2.56
Age 11	2992	17.21	2.49	2366	17.45	2.65	3162	17.38	2.79	2052	17.70	3.05
Age 12	3934	17.70	2.62	3062	17.90	2.88	4108	17.83	2.80	2980	17.98	2.97
Age 13	1198	18.41	2.94	1002	18.60	3.22	1124	18.85	3.23	834	18.91	3.19
Age 14	2072	19.16	2.73	1848	19.45	3.11	2410	19.47	3.00	1890	19.66	3.17
Age 15	1228	19.98	3.16	1094	20.20	3.17	1164	20.37	3.44	992	20.81	3.75
Age 16	1614	20.59	2.88	1550	20.78	2.97	1996	20.55	2.87	1700	20.80	3.11
Age 17	1824	21.11	2.80	1910	21.46	3.02	2464	20.69	2.87	1988	20.95	3.00
Age 18	2028	21.35	2.55	1694	21.89	2.92	1378	21.29	3.18	1140	21.44	3.32
Age 19	814	21.57	2.49	784	21.82	2.46	998	21.04	3.01	734	21.49	3.17
Age 20–29	2786	23.19	3.03	2290	23.45	2.96	2804	22.12	3.73	2118	22.15	3.51
Age 30–39	1242	24.78	3.34	1066	25.20	3.62	2114	22.94	4.05	1686	22.82	3.99
Age 40–49	670	26.11	3.48	492	26.54	3.95	1096	24.15	4.80	782	23.86	4.39

Names list of the participating twin cohorts in this study: Australian Twin Registry, Boston University Twin Project,^a,b^ Carolina African American Twin Study of Aging, Colorado Twin Registry,^b^ East Flanders Prospective Twin Survey,^b^ Finntwin12,^a,b^ Finntwin16,^a,b^ Gemini Study,^a,b^ Guinea-Bissau Twin Study,^a^ Hungarian Twin Registry,^b^ Italian Twin Registry,^a^ Japanese Twin Cohort,^a^ Longitudinal Israeli Study of Twins, Michigan Twins Study, Minnesota Twin Family Study,^b^ Minnesota Twin Registry,^b^ Mongolian Twin Registry,^b^ Murcia Twin Registry, Norwegian Twin Registry, Peri/Postnatal Epigenetic Twins Study,^a,b^ Qingdao Twin Registry of Children, Quebec Newborn Twin Study,^a,b^ Swedish Young Male Twins Study of Adults,^a,b^ Swedish Young Male Twins Study of Children,^a,b^ Twins Early Developmental Study,^a,b^ West Japan Twins and Higher Order Multiple Births Registry^a,b^ and Young Netherlands Twin Registry.^a,b^ All twin cohorts were used in the analyses on the association between birthweight and later BMI (total sample). ^a^Twin cohorts used in the analyses involving birth length/PI. ^b^Twin cohorts used in the analyses involving gestational age.

Names list of the participating countries (number of twin cohorts per country, % of the total sample): Australia (2, 0.51%), Belgium (1, 0.31%), Canada (1, 1.63%), China (1, 0.32%), Finland (2, 10.88%), Guinea-Bissau (1, 0.08%), Hungary (1, 0.06%), Israel (1, 0.29%), Italy, (1, 0.59%), Japan (2, 12.19%), Mongolia (1, 0.04%), Netherlands (1, 35.28%), Norway (1, 1.99%), Spain (1, 0.06%), Sweden (2, 4.60%), United Kingdom (2, 20.47%), USA (6, 10.69%).

In the original database, there were 122 582 twin individuals with information on birthweight. We excluded 81 individuals with birthweight < 0.5 or > 5 kg. The remaining 122 501 individuals presented a total of 355 650 height and weight measurements at later ages. Age was classified to single-year age groups from age 1 to 19 years (e.g. age 1 refers to 0.5–1.5 years range) and three adult age groups (20–29, 30–39 and 40–49 years). Measurements at ages ≤0.5 and > 49.5 years (which is a proxy for menopausal status in women) were excluded because the sample sizes were too small. BMI was calculated as weight (kg)/square of height (m^2^). Impossible values and outliers were checked by visual inspection of histograms for each age and sex group and were removed (< 0.3 % of the measurements) allowing the distribution of BMI data to be positively skewed, resulting in 344 104 measurements. After restricting the analyses to one BMI measure per individual in each age group by keeping the measurement at the youngest age (6% of the measurements were removed), we had 324 968 observations from 119 323 individuals.

We next excluded unmatched pairs (without data on their co-twins), resulting in 149 435 paired observations. Furthermore, because of the effects of sex differences within a pair on both birthweight and BMI especially during and after puberty, opposite-sex dizygotic twin pairs were excluded (41 733 paired observations). Intra-pair differences in birthweight and later BMI were checked by visual inspection of histograms. We removed birthweight differences greater than ±1.7 kg (72 paired observations) and outliers for the within-pair BMI difference in each age group (125 paired observations). Together, we had 214 930 observations (107 465 paired observations), 55% MZ and 45% same-sex DZ, from 78 642 twin individuals (39 321 complete twin pairs). In summary, after excluding opposite-sex dizygotic twin pairs, the study database (39 321 twin pairs) is 95% of the eligible sample (41 599 twin pairs).

For secondary analyses, we additionally calculated birthweight standardized by gestational age and ponderal index (PI) at birth. Birthweight was expressed as standard deviation (SD) scores of the respective means/weeks of gestation (*z*-scores; i.e. mean = 0 and SD = 1) to estimate the relative position of birthweight for a given gestational age. Individuals without data on gestational age, gestational age < 25 or > 45 weeks or with discordant information on gestational age within pairs were excluded. Unrealistic birthweight values for a given gestation were checked by visual inspection of histograms for each gestational week and removed (< 0.2% of the observations). After these exclusions, we had 84 357 paired observations. For the analyses on PI [weight (kg)/height (m^3^)], we removed those cases without information on birth length, birth length < 25 or > 60 cm, PI < 12 or > 38 or intra-pair difference in PI > 15 kg/ m^3^ (from the 107 465 paired observations in the primary analyses), resulting in 68 954 paired observations.

All participants were volunteers and they or their parents gave informed consent when participating in their original studies. Only a limited set of observational variables and anonymized data were delivered to the data-management centre at University of Helsinki. The pooled analysis was approved by the ethical committee of the Department of Public Health, University of Helsinki, and the methods were carried out in accordance with the approved guidelines.

### Statistical analyses

Statistical analyses were conducted using the Stata statistical software package (version 12.0; StataCorp, College Station, TX, USA). First, all BMI measurements were adjusted for exact age within each age and sex groups using linear regression (BMI was used as dependent variable and age as continuous independent variable) and the resulting residuals were used as input variables for the following analyses.

In primary analyses, we studied the association between birthweight and BMI residuals at both the individual and within-pair levels. At the individual level, linear regression models for each age, sex and zygosity group were used with birthweight as the explanatory variable and BMI residuals as the outcome. Associations were adjusted for birth year and twin cohort (treated as continuous and categorical, respectively). The non-independence within twin pairs was taken into account by using the ‘cluster’ option available in Stata. Since regression analyses with log-transformed BMI and untransformed BMI provided very similar results, we used untransformed BMI data in order to make these results comparable with those from the pair-wise analyses. In the within-pair analyses, intra-pair differences with both positive and negative values were created by randomly subtracting the co-twin with the lowest birthweight from the co-twin with the highest birthweight or vice versa. At the within-pair level, we performed linear regression models for each age, sex and zygosity group with intra-pair birthweight difference as the explanatory variable and intra-pair BMI residuals difference as the outcome. Associations were also adjusted for birth year and twin cohort. Next, we ensured that the regression lines passed through the origin by checking that the intercept was not different from zero.

An interaction analysis was performed to investigate whether zygosity influenced the associations between birthweight and BMI residuals by introducing a product term of zygosity and birthweight into the regression model. At the individual level, linear regression models for each age and sex group were used with birthweight as the explanatory variable and BMI residuals, zygosity, the product term of zygosity and birthweight, birth year and twin cohort as the regressors. At the within-pair level, linear regression models for each age and sex group were performed with intra-pair birthweight difference as the explanatory variable and intra-pair BMI residuals difference, zygosity, the product term of zygosity and intra-pair birthweight difference, birth year and twin cohort as the regressors. There was no interaction effects between zygosity and birthweight in individual-level analyses (only 2 of 44 tests had *P*-value < 0.05 and none of them had *P*-value < 0.0011 that would correspond to *P*-value < 0.05 after Bonferroni correction of multiple testing); similar findings were observed between zygosity and intra-pair birthweight differences in pair-wise analyses (Appendix Table 1). The quadratic effect of birthweight was investigated by introducing the term in the regression models for the association between birthweight and BMI residuals, i.e. by introducing the quadratic term of birthweight in the individual-level analyses and the quadratic term of intra-pair birthweight differences in the pair-wise analyses. No quadratic effect of birthweight or intra-pair birthweight differences was found (results on request).

In secondary analyses, we first analysed the association between birthweight standardized for gestational age and BMI residuals at the individual level. Linear regression models for each age, sex and zygosity group were used with gestational age-standardized birthweight as the explanatory variable and BMI residuals as the outcome. Associations were adjusted for birth year and twin cohort. Finally, we analysed the association between PI at birth and BMI residuals both at the individual and within-pair levels (also adjusted for birth year and twin cohort). At the individual level, linear regression models for each age, sex and zygosity group were used with PI as the explanatory variable and BMI residuals as the outcome. At the within-pair level, linear regression models for each age, sex and zygosity group were used with intra-pair PI difference as the explanatory variable and intra-pair BMI residuals difference as the outcome. Since all analyses were based on BMI residuals, we will refer, except in statistical methods section, to ‘BMI residuals’ as ‘BMI’ for simplicity.

## Results

Table 1 provides descriptive statistics for birthweight and BMI by zygosity, age and sex. Mean birthweight was slightly greater in males than in females and in DZ than in MZ twins; the same pattern was observed for the SD of birthweight. Regarding BMI, sample size for each zygosity, age and sex group ranged between 530 and 6176 measurements. The 6, 19 and 40–49 years age groups had the smallest sample sizes. Mean BMI declined from the age of 1 to 5 years and then started to increase; these mean values were higher in males than in females from age 1 to 6 years and from the age of 17 years onwards. The SD of BMI generally increased with age. Despite similar values in early childhood, DZ twins had slightly higher mean BMI and greater SD than MZ twins at most ages.

At the individual level, birthweight was generally positively associated with later BMI; regression coefficients showed that a 1-kg increase in birthweight was associated with up to 0.9 kg/m^2^ higher BMI, ranging between 0.3 and 0.6 kg/m^2^ at most ages (Table 2). The magnitude of the associations fluctuated more in adolescence and adulthood, probably explained by the smaller sample size, and no association was observed for some age-zygosity groups. When birthweight was expressed as a *z*-score for gestational age, the associations generally slightly increased in childhood and early adolescence. From middle adolescence onwards, the pattern was not clear, with some decreased associations in boys (Appendix Table 2).

**Table 2:** Regression coefficients for the association between birthweight and BMI (BMI units per kg birthweight), with monozygotic (MZ) and dizygotic (DZ) twins treated as individuals (individual level)

	Males								Females							
	MZ				DZ				MZ				DZ			
	B	*P*-value	95% CIs		B	*P*-value	95% CIs		B	*P*-value	95% CIs		B	*P*-value	95% CIs	
Age 1	0.52	<0.001	0.43	0.61	0.40	<0.001	0.32	0.48	0.43	<0.001	0.34	0.53	0.52	<0.001	0.43	0.61
Age 2	0.55	<0.001	0.46	0.65	0.50	<0.001	0.41	0.59	0.49	<0.001	0.39	0.60	0.56	<0.001	0.47	0.66
Age 3	0.53	<0.001	0.44	0.63	0.45	<0.001	0.36	0.53	0.45	<0.001	0.36	0.54	0.43	<0.001	0.33	0.53
Age 4	0.55	<0.001	0.40	0.69	0.42	<0.001	0.27	0.57	0.50	<0.001	0.34	0.67	0.51	<0.001	0.36	0.67
Age 5	0.56	<0.001	0.41	0.71	0.39	<0.001	0.24	0.53	0.49	<0.001	0.35	0.64	0.49	<0.001	0.34	0.65
Age 6	0.46	0.002	0.16	0.76	0.39	0.015	0.08	0.70	0.34	0.021	0.05	0.64	0.67	0.003	0.23	1.11
Age 7	0.32	<0.001	0.20	0.44	0.41	<0.001	0.29	0.54	0.45	<0.001	0.31	0.59	0.39	<0.001	0.25	0.54
Age 8	0.67	<0.001	0.52	0.83	0.40	<0.001	0.20	0.60	0.44	<0.001	0.23	0.64	0.63	<0.001	0.38	0.88
Age 9	0.40	0.001	0.17	0.63	0.61	<0.001	0.34	0.88	0.57	<0.001	0.33	0.81	0.55	0.002	0.21	0.90
Age 10	0.39	<0.001	0.22	0.56	0.40	<0.001	0.22	0.58	0.40	<0.001	0.21	0.59	0.37	<0.001	0.17	0.56
Age 11	0.55	<0.001	0.33	0.77	0.44	<0.001	0.20	0.69	0.41	0.002	0.15	0.66	0.54	0.001	0.24	0.85
Age 12	0.50	<0.001	0.30	0.70	0.51	<0.001	0.30	0.73	0.35	0.002	0.13	0.56	0.37	0.003	0.13	0.62
Age 13	0.19	0.358	–0.22	0.60	0.21	0.364	–0.24	0.66	0.16	0.480	–0.28	0.59	–0.19	0.448	–0.67	0.30
Age 14	0.36	0.012	0.08	0.65	0.30	0.065	–0.02	0.62	0.17	0.255	–0.12	0.46	0.13	0.395	–0.17	0.44
Age 15	0.20	0.329	–0.20	0.59	0.48	0.009	0.12	0.84	0.64	0.007	0.18	1.09	0.03	0.922	–0.48	0.53
Age 16	0.52	0.001	0.20	0.83	0.66	<0.001	0.29	1.03	0.62	<0.001	0.30	0.95	0.45	0.005	0.13	0.77
Age 17	0.33	0.030	0.03	0.62	0.71	<0.001	0.43	0.98	0.35	0.015	0.07	0.64	0.37	0.008	0.10	0.64
Age 18	0.28	0.046	0.00	0.55	0.02	0.911	–0.30	0.33	0.42	0.048	0.00	0.83	0.20	0.409	–0.28	0.68
Age 19	0.66	0.010	0.16	1.15	0.86	<0.001	0.52	1.20	0.86	0.001	0.33	1.38	0.38	0.141	–0.13	0.88
Age 20–29	0.41	0.003	0.14	0.69	0.48	<0.001	0.22	0.73	–0.07	0.687	–0.42	0.28	0.32	0.035	0.02	0.63
Age 30–39	0.55	0.005	0.17	0.94	0.93	<0.001	0.50	1.35	0.32	0.086	–0.05	0.69	–0.12	0.533	–0.49	0.26
Age 40–49	–0.08	0.745	–0.58	0.41	0.77	0.013	0.16	1.38	–0.06	0.837	–0.58	0.47	0.04	0.872	–0.49	0.57

Birthweight was used as the explanatory variable and BMI as the outcome. Associations were adjusted for birth year and twin cohort.

B, regression coefficient; 95% CIs, 95% confidence intervals.

**Figure 1:**
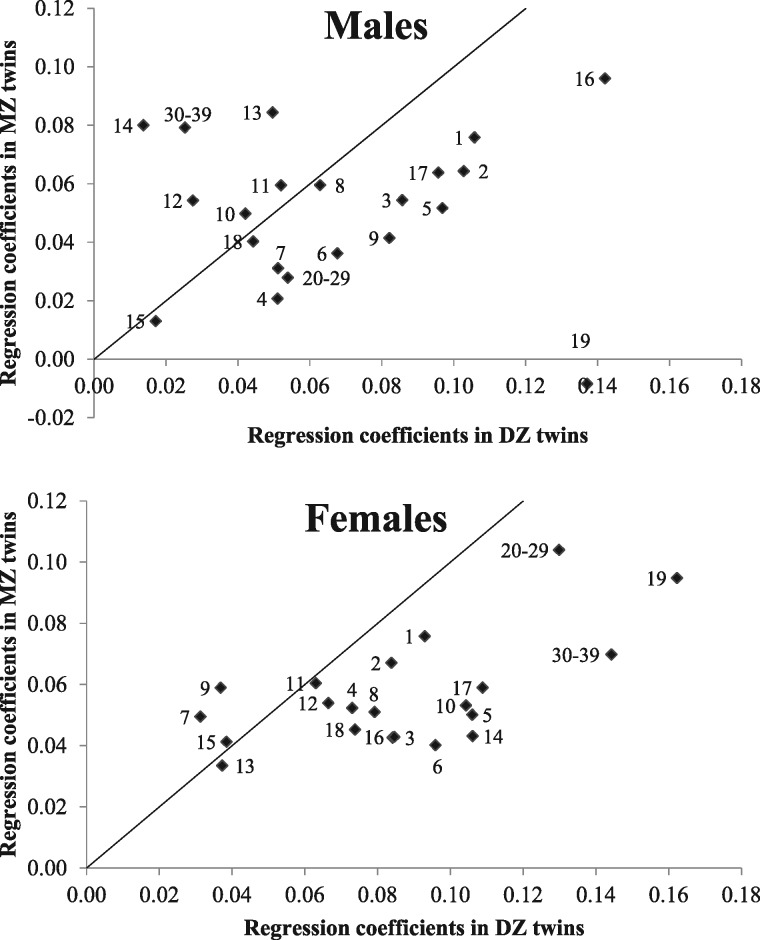
Scatter plots of the regression coefficients for the intra-pair differences in PI at birth and later BMI (BMI units per PI unit) in monozygotic (MZ) vs. dizygotic (DZ) twins. Plot labels indicate the specific age (years at BMI measurements) at which the associations were analyzed.

Within MZ twin pairs, greater birthweight was also associated with higher BMI at most ages (Table 3). Regression coefficients generally ranged from 0.6 to 1.0 kg/m^2^ per kg birthweight (up to 1.2 kg/m^2^), were similar in males and females, and somewhat greater in childhood than in late adolescence and adulthood; no association was observed at 40–49 years. Supported by the lack of interaction between zygosity and intra-pair birthweight differences, the magnitude of the associations in DZ twins was similar to that of MZ twins; when different, they were generally greater in MZ twins (except at 9 and 19 years in males). A positive association was also observed between PI at birth and later BMI (Figure 1 and Appendix Table 3). A MZ intra-pair difference of a 1-kg/m^3^ increase in PI generally resulted in a BMI difference of 0.03–0.08 kg/m^2^, but the effects were somewhat greater in DZ twins at some ages.

**Table 3:** Regression coefficients for the association between intra-pair differences in birthweight and BMI (BMI units per kg birthweight) in monozygotic (MZ) and dizygotic (DZ) twins (within-pair level)

	Males								Females							
	MZ				DZ				MZ				DZ			
	B	*P*-value	95% CIs		B	*P*-value	95% CIs		B	*P*-value	95% CIs		B	*P*-value	95% CIs	
Age 1	0.92	<0.001	0.84	0.99	0.88	<0.001	0.77	1.00	1.05	<0.001	0.98	1.13	0.97	<0.001	0.84	1.09
Age 2	0.84	<0.001	0.76	0.93	0.97	<0.001	0.84	1.09	0.97	<0.001	0.90	1.05	0.83	<0.001	0.69	0.96
Age 3	0.76	<0.001	0.69	0.83	0.78	<0.001	0.66	0.89	0.89	<0.001	0.82	0.97	0.80	<0.001	0.68	0.92
Age 4	0.71	<0.001	0.60	0.83	0.78	<0.001	0.61	0.96	0.87	<0.001	0.74	1.00	0.73	<0.001	0.53	0.94
Age 5	0.81	<0.001	0.69	0.92	0.91	<0.001	0.73	1.09	0.80	<0.001	0.69	0.92	0.90	<0.001	0.67	1.12
Age 6	0.79	<0.001	0.61	0.98	0.58	0.002	0.21	0.95	0.97	<0.001	0.74	1.20	1.01	<0.001	0.51	1.51
Age 7	0.70	<0.001	0.60	0.80	0.65	<0.001	0.48	0.83	0.98	<0.001	0.89	1.08	0.54	<0.001	0.35	0.73
Age 8	0.80	<0.001	0.66	0.94	0.89	<0.001	0.60	1.18	0.95	<0.001	0.81	1.09	1.07	<0.001	0.72	1.43
Age 9	0.72	<0.001	0.52	0.91	1.24	<0.001	0.83	1.65	1.08	<0.001	0.91	1.25	0.69	0.003	0.24	1.14
Age 10	0.83	<0.001	0.69	0.96	0.62	<0.001	0.36	0.88	1.06	<0.001	0.94	1.19	0.90	<0.001	0.60	1.21
Age 11	0.98	<0.001	0.80	1.15	0.79	<0.001	0.45	1.14	1.10	<0.001	0.94	1.26	0.98	<0.001	0.54	1.41
Age 12	0.83	<0.001	0.68	0.98	0.75	<0.001	0.44	1.06	0.97	<0.001	0.81	1.12	0.57	0.002	0.21	0.93
Age 13	1.05	<0.001	0.71	1.38	1.03	0.001	0.43	1.63	0.89	<0.001	0.53	1.25	0.63	0.087	–0.09	1.34
Age 14	0.87	<0.001	0.61	1.12	0.84	<0.001	0.39	1.29	0.71	<0.001	0.47	0.96	0.80	0.001	0.32	1.27
Age 15	0.78	<0.001	0.48	1.08	0.35	0.226	–0.22	0.92	1.05	<0.001	0.68	1.41	0.47	0.209	–0.27	1.21
Age 16	0.85	<0.001	0.53	1.16	1.05	<0.001	0.52	1.58	0.73	<0.001	0.46	0.99	0.86	0.002	0.33	1.39
Age 17	0.48	0.001	0.20	0.76	0.54	0.027	0.06	1.02	0.64	<0.001	0.37	0.90	0.75	0.002	0.27	1.22
Age 18	0.60	<0.001	0.37	0.83	0.22	0.367	–0.26	0.71	0.96	<0.001	0.60	1.33	0.88	0.011	0.20	1.55
Age 19	0.17	0.447	–0.27	0.61	0.84	0.012	0.18	1.50	0.75	<0.001	0.36	1.15	0.96	0.018	0.17	1.75
Age 20–29	0.41	0.002	0.16	0.67	0.38	0.079	–0.04	0.80	0.68	<0.001	0.35	1.02	0.48	0.071	–0.04	0.99
Age 30–39	0.27	0.239	–0.18	0.72	0.73	0.041	0.03	1.44	0.50	0.018	0.09	0.92	0.51	0.139	–0.17	1.20
Age 40–49	–0.15	0.615	–0.73	0.43	–0.20	0.740	–1.40	1.00	0.11	0.739	–0.54	0.76	–1.10	0.044	–2.18	–0.03

Intra-pair birthweight difference was used as the explanatory variable and intra-pair BMI difference as the outcome. Associations were adjusted for birth year and twin cohort.

B, regression coefficient; 95% CIs, 95% confidence intervals.

## Discussion

The present study, based on a multinational database of 27 twin cohorts with 107 465 paired observations, showed that birthweight is associated with later BMI in males and females from infancy onwards, but the association tends to be attenuated in adulthood. Because the associations are observed within MZ pairs, our results support the role of environmental factors unique to each individual in the relationship and refine previous findings by considering, in addition to adult age, childhood and adolescence using 1-year age groups from 1 to 19 years.

At the individual level, the increase in BMI associated with a 1-kg increase in birthweight (0.3–0.6 kg/m^2^ at most ages) was in the range of other twin and singletons studies in late adolescence and young adulthood.^4^^,^^9^^,^^18^ The quadratic effects of birthweight were independently tested in each age, zygosity and sex groups, and there was no evidence of non-linearity between birthweight and later BMI. Further, since smallness for gestational age, rather than smallness due to prematurity, has shown to be an indicator for shortness and lightness in early childhood,^23^ we standardized birthweight for gestational age. The magnitude of the associations slightly increased until early adolescence, suggesting that the effect of gestational age on the association between birthweight and BMI remains important, at least until this period.

The pair-wise analysis of MZ twins showed that environmental individual-specific factors are important in the association between birthweight and later BMI, suggesting the role of the intrauterine environment. The magnitude of these individual-specific factors tended to persist during childhood but decreased from late adolescence. For example, the effects at ages 20–29 years (0.41 kg/m^2^ and 0.68 kg/m^2^ per kg in males in females, respectively) were comparable with those reported in other studies^18–21^; however, none of them analysed the relationship in childhood. These intra-pair associations between birthweight and later BMI observed in different populations suggest that a causal relation is biologically plausible. The number of fat cells (adipocytes) has shown to be a major determinant of fat mass in adults.^24^ Spalding *et al.*^24^ found that the adipocyte number is set during childhood and adolescence and, although there is a high turnover (10% annually), stays constant during adulthood. Further, there is evidence that the number of muscle cells in the body is determined before birth.^25^ Since intra-pair differences in birthweight have shown a positive association with intra-pair differences in both total lean mass and total fat mass,^26^ one possible explanation is that higher birthweight implies a greater number of cells in both adipose and non-adipose tissues, and this cell number difference remains in later life. The decreasing association between birthweight and BMI across adulthood might be explained by changes in BMI independently of the number of fat or muscle cells, but also by a lower accuracy of birthweight measurements in individuals born earlier (69% of the individuals with BMI measurements at 40–49 years born before 1950).

There is also evidence that environmental exposures during early life can induce persistent alterations in the epigenome, which may lead to an increased risk of obesity later in life.^27^ For example, a recent study suggested that both maternal obesity and, to a larger degree, underweight affect the neonatal epigenome via an intrauterine mechanism.^28^ DNA methylation patterns in cord blood showed some association with altered gene expression, body size and composition in childhood, but the authors found no association between methylation status and birthweight.^29^ A twin study using gene expression discordance as a proxy measure of epigenetic discordance in MZ twins at birth reported some association between birthweight and expression of genes involved in metabolism and cardiovascular function.^30^ However, there is no evidence, to our knowledge, of epigenetic mechanisms explaining the positive association between birthweight and later BMI. It is noteworthy that overall epigenetic changes are weakly associated with BMI and are more prominent only when metabolic complications of obesity arise.^31^

Although the findings from previous studies are contrasting,^18^^,^^20^^,^^21^ our data revealed that the magnitude of the associations in DZ pairs was generally similar to that in MZ pairs and thus suggest that genetic factors are not very importantly involved in the relationship between birthweight and later BMI. This is supported by a recent study using linkage-disequilibrium score regression, which estimated a genetic correlation of 0.11 between birthweight and adult BMI.^32^ However, in the absence of data on chorionicity, a possible genetic influence cannot be fully excluded. Approximately two-thirds of MZ twins are monochorionic and thus share the same placenta; an unequal placental sharing is a major cause of fetal growth discordance in MZ twins.^33^ Therefore, intrauterine factors that could potentially account for our findings are placental differences between MZ and DZ twins and between monochorionic and dichorionic MZ twins.^33^^,^^34^ It has been reported that monochorionic MZ twins are more discordant than dichorionic MZ twins for BMI throughout childhood and adolescence.^33^ Therefore, it could be argued that, besides genetic factors, these placental differences may increase the intra-pair associations in MZ pairs, making them thus more similar to those in DZ pairs.

Birthweight may not be the ideal measurement of body composition in newborns, since it does not discriminate between those infants of different sizes or body shapes. Thus, we repeated the analyses for PI, a measure of relative weight at birth. The effects were greater in DZ twins at some ages, suggesting that genetic factors may play a role in the association, which is agreement with the findings in Finnish twins.^21^ After standardization (to *z*-scores), the units of weight and PI at birth became comparable. It was then evident that intra-pair differences in BMI were more strongly associated with birthweight than with PI in most zygosity, age and sex groups (results not shown). However, neither PI nor BMI determine fat mass per se. BMI is generally used as a proxy for body fat in epidemiologic studies, but it does not allow the drawing of conclusions about body composition.^35^ As reviewed by Rogers,^10^ birthweight is usually positively associated with lean body mass and negatively associated with relative adiposity, suggesting that the association between birthweight and BMI/overweight does not necessarily reflect increased adiposity at higher birthweights.

The main strength of the present study is the large sample size of our multinational database of twin cohorts with information on size at birth and height and weight measures from infancy to adulthood. We performed an individual-based pooled analysis to provide results for this sample including the large majority of existing twin cohorts. Generalization for the global population is, however, not possible because countries or regions are not equally represented and the database is heavily weighted towards Caucasian populations following Westernized lifestyle. Another limitation of the data is that most of the measures were parentally reported (birth measures) and self-reported or parental-reported (later measures).^22^ However, the accuracy between maternal recall and medical records of birthweights (in singletons) have reached a kappa value of 0.89,^36^ and the correlations between measured and self-reported heights and weights have commonly been over 0.90.^37^^,^^38^ Finally, it has been questioned whether differences in birth size in twins are a suitable model for differences in birthweight in general, because intrauterine growth in twins is different from that in singletons and fetal growth may be particularly compromised in MZ twins.^39^ However, the magnitude of the relationship between birthweight and BMI in twins was at the same level as that reported in singletons.^4^ As concluded by Morley,^39^ there is no reason to suggest that data from twins cannot be used to shed light on causal pathways underlying the association between birthweight and cardiovascular risk factors.

In conclusion, our findings showed that environmental factors unique to each individual are important in the association between birthweight and later BMI, and thus support the role of the intrauterine environment in the development of later BMI. The association of birthweight with later BMI persists across ages but is attenuated in adulthood. Identifying intrauterine environmental factors affecting later BMI may thus be important when trying to understand the development of obesity across the life-span.
